# Crosstalk of Exosomal Non-Coding RNAs in The Tumor Microenvironment: Novel Frontiers

**DOI:** 10.3389/fimmu.2022.900155

**Published:** 2022-05-19

**Authors:** Zimo Jia, Jinlin Jia, Lihui Yao, Zhihan Li

**Affiliations:** ^1^Department of Biochemistry and Molecular Biology, Hebei Medical University, Shijiazhuang, China; ^2^The Second General Surgery, The Fourth Hospital of Hebei Medical University, Shijiazhuang, China; ^3^National Research Institute for Family Planning, National Human Genetic Resources Center, Beijing, China; ^4^Graduate School, Peking Union Medical College, Beijing, China; ^5^Department of Otolaryngology, Henan Province Hospital of Traditional Chinese Medicine, Zhengzhou, China

**Keywords:** exosomes, tumor microenvironment, non-coding RNAs, tumor immunity, cancer

## Abstract

The tumor microenvironment (TME) is defined as a complex and dynamic tissue entity composed of endothelial, stromal, immune cells, and the blood system. The homeostasis and evolution of the TME are governed by intimate interactions among cellular compartments. The malignant behavior of cancer cells, such as infiltrating growth, proliferation, invasion, and metastasis, is predominantly dependent on the bidirectional communication between tumor cells and the TME. And such dialogue mainly involves the transfer of multifunctional regulatory molecules from tumor cells and/or stromal cells within the TME. Interestingly, increasing evidence has confirmed that exosomes carrying regulatory molecules, proteins, and nucleic acids act as an active link in cellular crosstalk in the TME. Notably, extensive studies have identified non-coding RNAs (ncRNAs), including long non-coding RNAs (lncRNAs), microRNAs (miRNAs), and circular RNAs (circRNAs), that could be encapsulated by exosomes, which regulate the coordinated function within the TME and thus participate in cancer development and progression. In this review, we summarize recent literature around the topic of the functions and mechanisms of exosomal ncRNAs in the TME and highlight their clinical significance.

## Introduction

Tumors are stubbornly described as a simply accumulation of oncocytes. However, it is rather a heterogeneous collection of tumor, immune, stromal cells, various signaling molecules, and luxuriant extracellular matrix (ECM), namely the tumor microenvironment (TME) (1). TME refers to the complex and plentiful multicellular environment shaped by tumor cells that is permissive for their survival and growth. During the early stages of neoplasia, a vibrant interactive partnership emerges between tumor cells and constituents of the TME that renders tumor cell local infiltration and distant dissemination possible. In an attempt to conquer hypoxic, a prominent element compared to the natural inherent environment, TME has organized an accelerated angiogenesis operation to maintain the supply of oxygen and nutrients and to clear metabolic waste products. Nowadays, the paradigm of cancer research and treatment has shifted from a tumor-centered model to a TME-centered model.

As a subtype of extracellular vesicles (EVs)—a group of membranous vesicles shed from the plasmalemma with encapsulated particles—exosomes are characterized by a lipid bilayer membrane extracellular structure with an endosomal origin (2). In addition to proteins and DNAs, exosomes generally encompass lipids, mRNAs, as well as non-coding RNAs (ncRNAs) (3). Although exosomes share common characteristics and structures, the molecular constitutions of exosomes secreted by different cell types vary extraordinarily. As a robust messenger of intercellular communication, exosomes play a pivotal role in mediating cancer progression. Exosomes released by tumor cells participate in substance transmission and information exchange *in vivo* and serve as the benchmark for cancer development and progression (4). Moreover, exosome biology and its multifaceted functions in the TME have drawn increasing attention in recent years. Of note, with the advanced proceedings in ncRNA sequencing methodologies, the special characters of exosomes in the TME are increasingly elicited, which has provoked tremendous interest in this research field.

ncRNAs are mainly subdivided into three main categories, namely: long ncRNAs (lncRNAs), microRNAs (miRNAs), and circular RNAs (circRNAs) (5). Until recently, the prevalence of ncRNAs in intercellular communication has been demonstrated by various investigations, thus shedding light on the complex crosstalk between cancer cells and non-cancer cells. On one hand, ncRNAs represent one of the most informative and significant components of all exosomal cargos (6). On the other hand, ncRNAs are increasingly being recognized as key regulators involved in the TME (7). Furthermore, tumor-derived exosomes (TDEs) contain a considerable proportion of ncRNAs, which are implicated in modulating cellular interplays and communication in the TME. Recent findings have strongly pinpointed that the dysfunction of global exosomal ncRNAs abundance is correlated with cancer immunity, metastasis, angiogenesis, drug resistance, and cancer stem cell (CSC) stemness (8–11). The multiple roles of exosomal ncRNAs in the TME explain their potential emerging clinical implications for developing novel treatment applications. Additionally, the exosomal ncRNAs display a variety of characteristics, including high abundance, relative stability, and cross-species conservation. And these features are critical considering the current worldwide endeavors to determine ncRNA signatures in tumor biopsy tissues using high-throughput sequencing technologies to develop effective diagnostic and prognostic biomarkers.

Nevertheless, the underlying mechanism of exosomal ncRNAs in regulating the TME is still unclear, warranting further exploration. Therefore, uncovering the exact functions and underlying regulatory mechanisms of exosomal ncRNAs in the TME is essential for optimizing current therapeutic strategies. Our previous study concluded the functions and mechanisms of ncRNAs in colorectal cancer (CRC) (12). This review aims to summarize the interactions between exosomal ncRNAs and TME. Also, the promising clinical significance of exosomal ncRNAs in tumor immunotherapy has been discussed in this paper.

## Biogenesis and Characteristics of Exosomes

**Figure 1:**
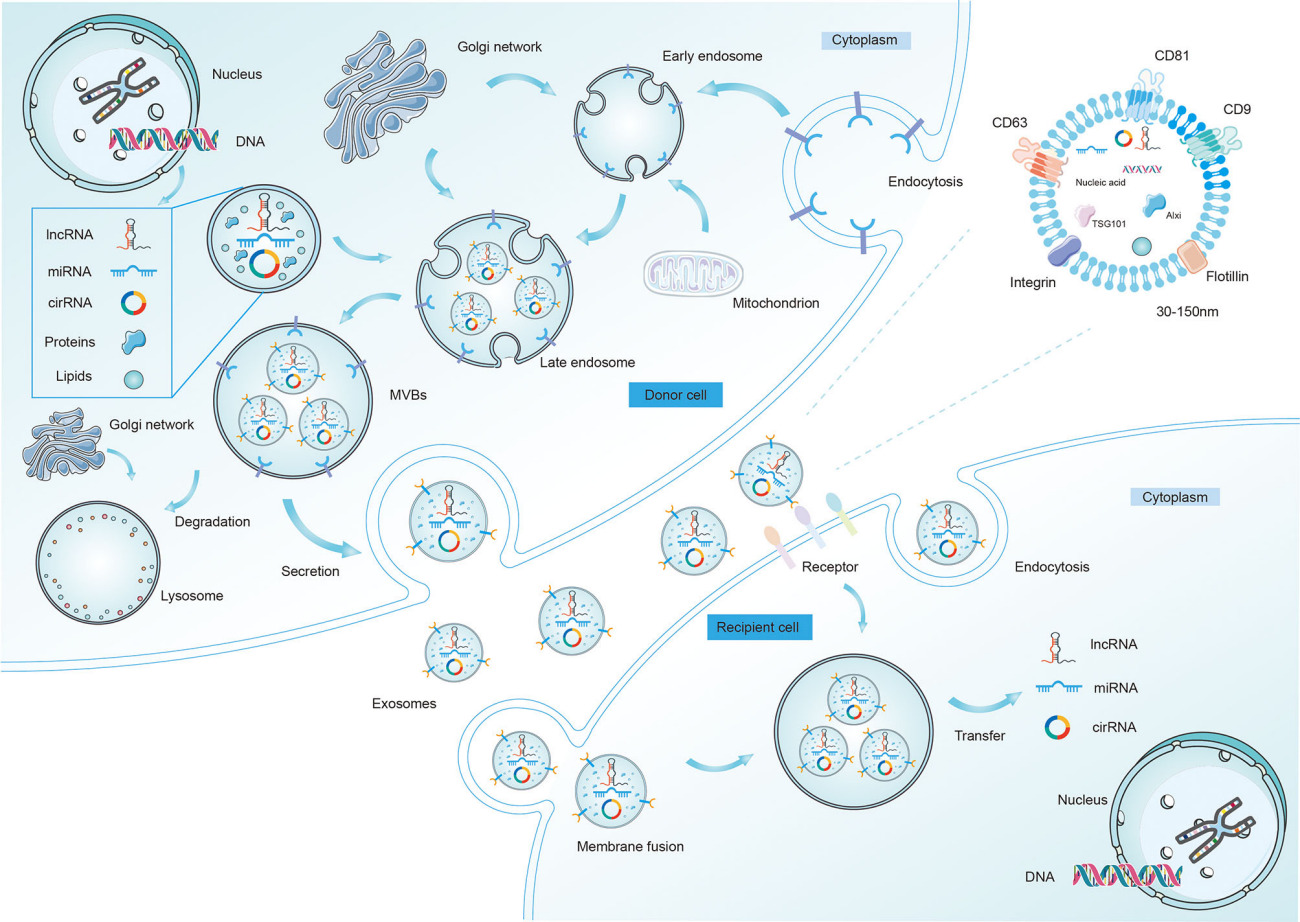
The biogenesis, release, and characteristics of exosomes. The formation and release of exosomes is a multi-step process. First, it begins with early endosome formation through endocytosis of the plasma membrane, followed by formation of the late endosome with different ncRNAs, lipids, and proteins inside. Second, the late endosomes undergo multiple depressions and bud inward to form microvesicles, thus transforming into multi-vesicular bodies (MVBs), which then may be degraded by lysosomes or fuse with the cell membrane and are released into the extracellular space as exosomes. After being secreted from donor cells into extracellular matrix, exosomes can be taken up by recipient cells and regulate the behaviors of the recipient cells by transferring regulatory non-coding RNAs (mainly miRNAs, lncRNAs, and circRNAs). Exosomes enter recipient cells in three ways, which are fusion, endocytosis, and receptor interactions. Several proteins are used as markers for exosomes (CD9, CD63, CD81, flotillin, integrin). Exosomes (30-150nm) also contain different intracellular proteins (such as TSG101 and Alix), RNAs, DNAs, and metabolites.

Exosomes are nanoscale vesicles with a size ranging from 30–150 nm (13). Current knowledge has confirmed that exosomes are formed by dynamic multistep endocytosis pathways (14). A natural course of exosome formation is composed of the following procedures (**Figure 1**). First, the cytoplasmic membrane invaginates to materialize endocytic vesicles, which in turn form early secretory endosomes. Subsequently, with the assistance of the Golgi complex, early endosomes undergo some changes and gradually mature into late endosomes (15). Then, in a series of finely modulated maturation processes, membrane-enclosed ILVs are produced by the inward budding of late endosomal compartments limiting membrane. Different types of cargo are sorted into ILVs during this procedure. Thereafter, this structure is referred to as an MVB, which is then carried to the cell surface along protein tracks. Then, the MVBs fuse with either the lysosome (where the ILVs are disintegrated) or the plasma membrane, activating the discharge of the vesicles inside the MVBs (16). Once these vesicles have been released by fusion with the plasma membrane, they eventually become known as exosomes. Notably, during the period of exosome biogenesis, various surface proteins and molecules (e.g., CD9, CD63, CD81, integrin, and flotillin) that are characteristics of the parent cells are selectively expressed on released exosomes.

As previously mentioned, exosomes contain diverse cargos, including proteins and RNA molecules. Most recently, exosomal RNA cargoes have been broadly investigated as to their crucial roles in intercellular communication during TME evolution. Accumulating evidence has indicated a conspicuous discordant enrichment distribution of RNAs in exosomes owing to the intrinsic sorting mechanism that aggressively manipulates the exosomal loading of certain RNAs. And these machineries appear to be based on RNA binding proteins (RBPs) and their affiliated companions, which dispatch RNAs to predetermined locations of exosome formation and shield RNAs from damage or destruction (17). The present research mainly concentrates on the regulatory factors of miRNA partitioning into exosomes. The enrichment of intracytoplasmic miRNAs is likely to be the first layer to bear the brunt since the presence of the miRISCs complex (main components within it are AGOs and GW182), which possesses the ability to drive the RNA-silencing process and affect miRNA pool placement (18). Interestingly, AGO2 can be passively incorporated into exosomes and therefore fine-tune the sorting of specific miRNAs (e.g., let-7a), revealing that AGO2 acts as an imperative transporter for exosomal miRNAs (19). Nonetheless, the biological significance of this voluntary sorting process in lncRNAs and circRNAs is not yet fully understood.

These secretory exosomes can be transported into adjacent or distant cells to mediate intercellular interactions by transferring regulatory ncRNAs or other functional molecules (20). Exosomes that reach other cells can function by binding to protein receptors on the cell surface. For one thing, exosomes directly perceive surface molecules located on the target cell, inducing signal transduction through receptor-ligand interactions. For another, *via* fusing with the plasma membranes of recipient cells, exosomes can also emit their contents into the cytosol, leading to changes in cellular functions. Additionally, receptor cells are able to take up exosomes through endocytosis. It has been shown that exosomes can preserve their cargo (including three main ncRNA species) from enzymatic degradation in plasma and bile. Correspondingly, exosomal ncRNAs are more stable, so they can exert stronger effects on recipient cells through their actions on target genes and pathways compared to those ncRNAs directly released into the extracellular space. To illustrate, lncUCA1 showed a discordant profile in serum exosomes and homologous cancerous tissues. Specifically, in CRC, the abundance of UCA1 was detected to be downregulated in TDEs while upregulated in CRC biopsies (21). This conflicting finding might be explained by the fact that UCA1 was cherished by CRC cells, which impelled the preservation of this lncRNA by tumor cells by reducing its release through exosomes. Another thing is that such a differential distribution tendency between primary cancers and exosomes might be the result of asymmetric interactions and ncRNA crosstalk among TME cells (22). Despite intensive studies on the biogenesis and characterization of exosomes, however, very little is known about their biological roles in exosome-secreting cells. As such, systematic experimental research is currently needed to elucidate the detailed functions and intrinsic sorting mechanisms of exosomal ncRNAs.

## The Inherent Characteristics and Functions of the TME

**Figure 2:**
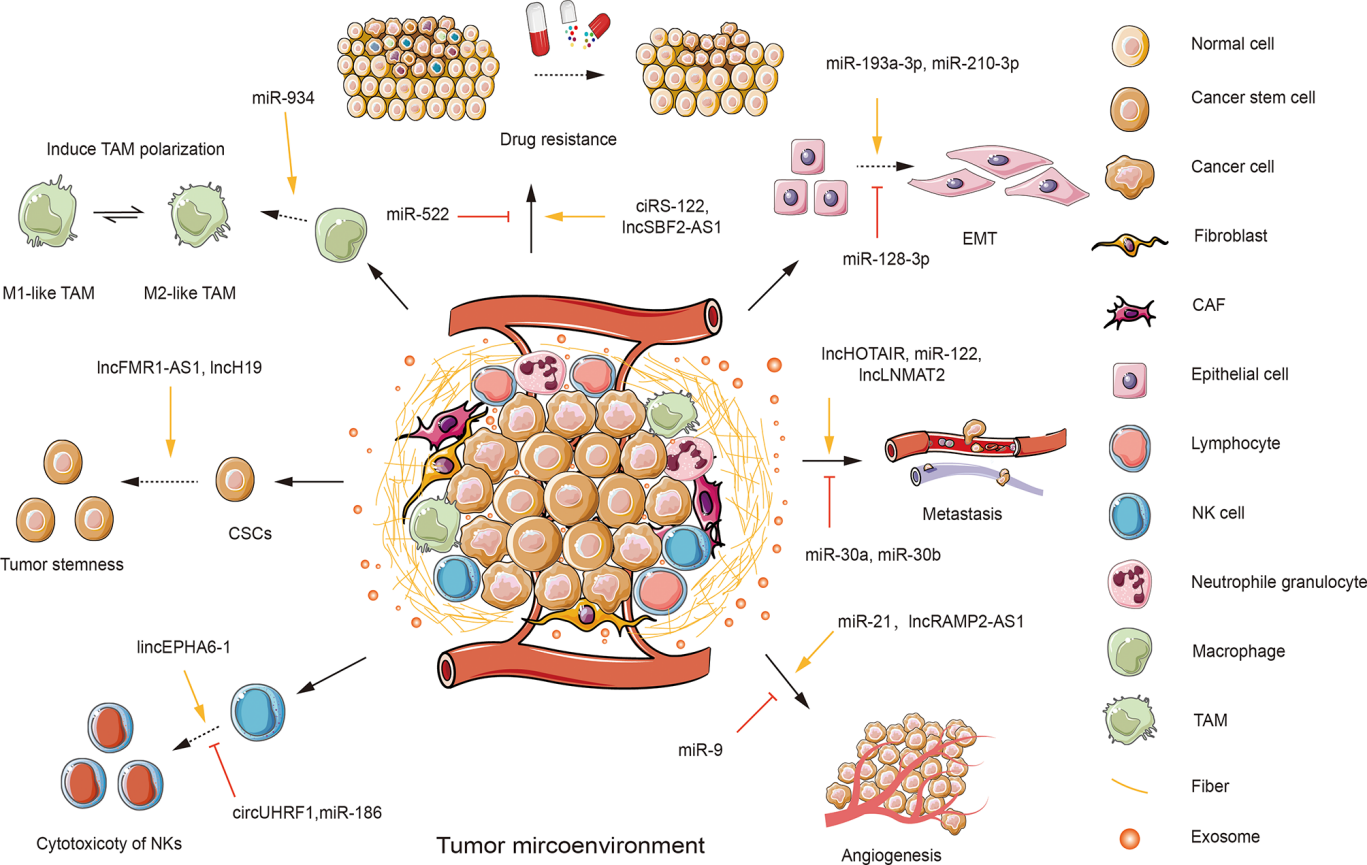
An overview of the roles of exosomal ncRNAs in the tumor microenvironment (TME). TME is composed of various components, including stromal cells, such as fibroblasts; immunity cells, such as T and B lymphocytes, natural killer cells (NKs), neutrophile granulocytes, macrophages; cancer cells; cancer stem cells (CSCs); cancer-associated cells, such as cancer-associated fibroblasts (CAFs) and tumor-associated macrophages (TAMs); and other infiltrated biomolecules, such as exosomes. Tumor cells could transfer functional exosomal ncRNAs to regulate the behaviors of recipient cells in the TME. Tumor-derived exosomal miR-934 can induce the polarization of TAMs. Several exosomal ncRNAs can suppress the cytotoxicity of NKs (e.g., circUHRF1 and miR-186). While some exosomal ncRNAs are shown to enhance the cytotoxicity of NKs (e.g., lincEPHA6-1). Some exosomal ncRNAs (e.g., lncFMR1-AS1 and miR-454) can enhance tumor stemness. Additionally, a few exosomal ncRNAs are involved in the regulation of tumor progression. Some exosomal ncRNAs can promote (e.g., miR-21 and lncRAMP2-AS1) or inhibit tumor angiogenesis (e.g., miR-9). Tumor-derived exosomes can transfer exosomal ncRNAs to induce (e.g., lncHOTAIR and miR-122) or suppress (e.g., miR-30a and miR-30b) tumor metastasis. Many exosomal ncRNAs play a role in facilitating (e.g., miR-193-3p and miR-210-3p) or repressing (e.g., miR-128-3p) epithelial-mesenchymal transition (EMT). Several exosomal ncRNAs are involved in promoting tumor drug resistance (e.g., ciRS-122 and lncSBF2-AS1).

The TME is a complex and dynamic ecosystem involved in various stages of tumorigenesis. It plays a multifaceted role in driving tumor initiation, promotion, propagation, and metastasis. And such involvement relies on intercellular interactions between cancer and non-cancer cells, where the trajectory of cancer greatly depends on the spatiotemporal communication between various cell types. It is, therefore, essential to more thoroughly deconstruct the complexity of the TME and to identify how TME cells crosstalk with each other to create an environment favorable for tumor progression. The TME is composed of assorted ingredients, including immune cells [e.g., various types of lymphocytes, natural killer cells (NKs), regulatory T cells (Tregs), and dendritic cells (DCs)], stromal constitutions (e.g., fibroblasts and fibers), tumor cells, myeloid-derived suppressor cells (MDSCs), cancer-associated fibroblasts (CAFs), and tumor-associated macrophages (TAMs), as well as other infiltrated biomolecules (23) (**Figure 2**). It is noteworthy that prior studies predominantly focused on tumor cells. But cancerous cells are not alone, as TME functions as a necessary determinant in altering both cancer cells and the surrounding stroma during cancer occurrence and development (24). This phenomenon gives rise to a mode of conversion in which carcinoma is currently recognized not only as a disorder defined by molecular (epigenetic and inherited) events within the cells, but also as an “ecological disease” manipulated by TME components (2).

The exchange and interaction of information within the TME have a significant impact on cancer initiation and progression, such as angiogenesis, metastasis, invasion, and other aggressive malignant behaviors of tumors (25). These tumor-influencing factors are largely dependent on TDEs excreted into the tumor stroma at the primary tumor site (26). Simultaneously, TDEs also impact non-cancerous cells, leading to the creation of a more aggressive TME that is conducive to tumor growth and metastatic spread (27). Numerous lines of literature imply that ncRNAs are selectively sorted into exosomes, which can be delivered and transferred between cells and regulate the initiation and development of cancer (4). More importantly, it has been confirmed that exosomal ncRNAs are one of the most decisive factors in cancer progression. They are involved in modulating cancer angiogenesis and metastasis, manipulating cancer chemoresistance, dominating host immune responses, and remodeling the cells in the TME (**Figure 2**).

TDEs can affect the functions of endothelial cells (ECs), thus inducing tumor angiogenesis and metastasis (28). In addition, TDEs are available to stimulate fibroblasts to differentiate into pro-tumorigenic CAFs and pro-angiogenic fibroblasts (29). Mechanistic studies revealed that it is cancer cell-derived exosomal ncRNAs that control these phenotypic switches. To further complicate matters, TDEs can initially inhibit immune cells, such as T cells, B cells, DCs, NKs, and so on. Subsequently, TDEs shape a T cell-absent immunosuppressive microenvironment to evade surveillance by excluding T cells from the neoplastic site or shifting immune cells into pro-tumorigenic or pro-metastatic phenotypes as the tumor progresses (30). And such phenotypic changes in immune cells are attributed to the transmission of functional oncoproteins from TDEs to recipient cells. Distinctively, these exosomal oncoproteins initiate MAPK and PI3K/AKT/mTOR pathways, facilitating tumorigenesis and metastasis (31). Further functional studies revealed that exosomal miR-934 markedly disturbs or impairs the functions of effector cells, including enhancing the enlargement trend and the immunosuppressive capacity of MDSCs, stimulating macrophages to polarize toward an M2-like phenotype, and inhibiting lymphocyte infiltration through matrix remodeling mediated by CAF (30). Growing evidence has verified that TDEs are indispensable for regulating cancer immunity, either through immune suppression or immune escape (32). For instance, glioma cells can shape and remodel immune cells by secreting exosomes. And these glioma-associated immune cells manage to control host immune responses and create an optimal environment for tumor survival and growth, thereby accelerating tumor development (33, 34).

## The Functions of Exosomal ncRNAs in the TME

Adequate comprehension of the molecular mechanisms by which cancer-associated immunity influences the evolution of the TME is one of the priorities of immunological research, which may facilitate the development of more potent immunotherapies. In light of previous studies, cancer immunity is a double-edged sword in cancers, where it either protects the host or promotes tumorigenesis depending on the tissue specificity. Tumor cells create a favorable environment for themselves by releasing a series of regulatory cytokines, chemokines, and other factors that modify or recode the surrounding immune cells. The spatiotemporal dialogue between tumor cells and proximal immune cells eventually culminates in an aggressive TME that accelerates cancer cell local invasion and metastatic spread. The characteristics of exosomes in the immune response during cancer evolution partly depend upon where they come from. Because exosomes from immune cells or tumors have different compositions, which can exert distinct biological effects. Exosomes from immune cells, such as those secreted by DCs, can induce T helper 1 (Th1) cell responses (e.g., IFN-γ production) in culture (35). Exosomes shed by DCs pulsed with ovalbumin (OVA) are more effective than micro-vesicles in stimulating activation of antigen OVA-specific CD8+ T cells (36). Activated T cells have been shown to initiate the host immune system, thereafter generating an anti-tumor immune response capable of limiting tumor growth and metastasis. In general, immune cells actively interact with each other by spontaneously producing exosomes. However, in most investigations, they are stromal cell receivers of TDEs. The ncRNAs-containing TDEs are information carriers that can alter or reprogram the functions of immune cells such as various T cells, DCs, macrophages, NKs, and MDSCs, thereby disrupting the immune responses of the host (37).

**Figure 3:**
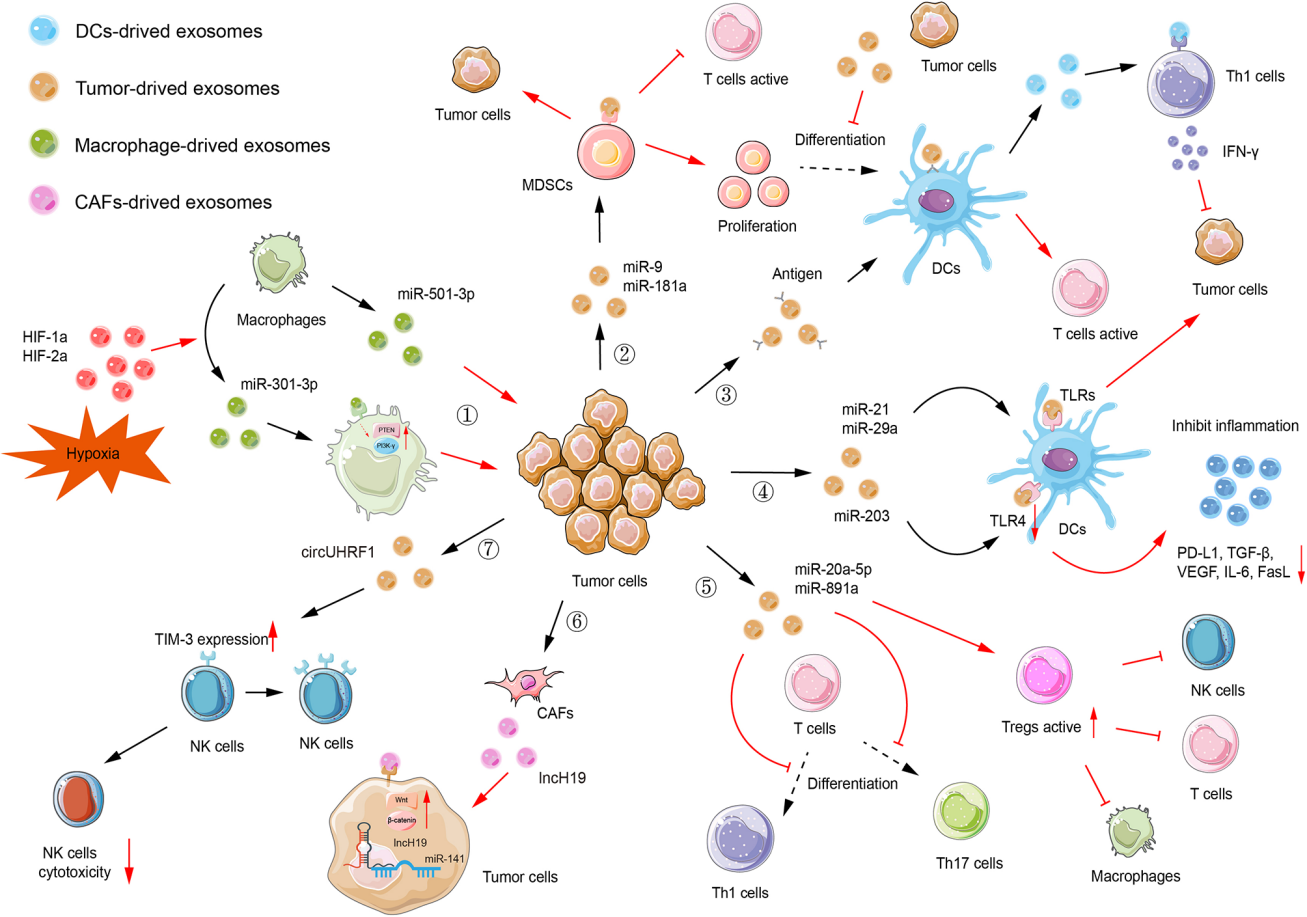
Exosomal ncRNAs-mediated immune regulation. (1) Macrophages-derived exosomal miR-501-3p facilitates tumor invasion and metastasis. Hypoxia can upregulate miR-301a-3p expression by modulating HIF-1a and HIF-2a, thus inhibiting the PTEN/PI3K-γ signaling pathway and promoting tumor invasion, migration, and epithelial-mesenchymal transition (EMT). (2) Tumor-derived exosomal miR-9 and miR-181a consequently affect proliferation of myeloid-derived suppressor cells (MDSCs) to promote tumorigenesis. Also, these MDSCs inhibit the activation of T cells. (3) Antigens can be encapsulated in exosomes and delivered to dendritic cells (DCs). Then, DCs activate T cells to initiate the immune response. Tumor-derived exosomes can inhibit the differentiation of MDSCs into DCs. DCs-derived exosomes facilitate the release of IFN-γ, which subsequently inhibits tumorigenesis. (4) Tumor-derived exosomal miR-21 and miR-29a can target TLRs to provoke pro-tumoral inflammation, rendering tumor growth and metastasis. Tumor-derived exosomal miR-203, targeting TLR4 in DC cells, downregulated the expression of TLR4 and related cytokines, such as PD-L1, TGF-β, and VEGF. (5) Tumor-derived exosomal miR-20a-5p and miR-891a inhibit Th1 and Th17 cell differentiation while increasing the activity of regulatory T cells (Tregs). (6) CAF-derived exosomal lncH19 can be transferred into tumor cells to activate Wnt/β-catenin signaling *via* serving as a ceRNA for miR-141. (7) Tumor-derived exosomal circUHRF1 can elevate the expression of TIM-3 on the surface of NKs, thus inhibiting the cytotoxicity of NKs.

The topic of exosomal ncRNAs in cancer immunity has evolved at an impressive rate in the past few decades, rendering it challenging or impractical to collect every single exosomal ncRNA in one review article. In what follows below, we concentrate predominantly on the most recent and paramount examples to expand the landscapes of representative exosomal ncRNAs in the TME. The regulation and mechanisms of several selected exosomal ncRNAs in immune cells are shown in **Figure 3**. Additionally, in **Table 1**, we summarize the functions and mechanisms of prominent exosomal ncRNAs in TME.

**Table 1:** Exosomal ncRNAs in cancer immunity.

Exosomal ncRNAs	Expression	Parent cell	Target cell	Biological function	Involved molecules or signaling pathways	Ref.
**TDEs**						
circUHRF1	↑	HCC	NKs	Inhibit NKs-derived IFN-γ and TNF-α secretion and promote metastasis	miR-449c-5p/ TIM-3	(38)
circUSP7	↑	NSCLC	CD8+ T cells	Induce CD8 + T cell dysfunction and anti-PD1 resistance	miR-934/SHP2	(39)
circ_0004658	↓	RBPJ-overexpressed macrophages	HCC	Inhibit tumor proliferation and migration	miR-499b-5p/JAM3	(40)
circ_0048117	↑	ESCC	M2 macrophages	Promote M2 macrophage polarization	miR-140	(41)
miR-19b-3p	↑	LCAD	M2 macrophages	Promote tumor metastasis and M2 macrophage polarization	PTPRD/STAT3	(42)
lncCRNDE-h	↑	CRC	CD4+T cells	Induce Th17 cells differentiation, increase the RORγt expression and IL-17 activity while inhibiting tumor growth	PPXY	(43)
lncDLX6-AS1	↑	HCC	M2 macrophages	Promote M2 macrophage polarization, tumor migration and invasion	miR-15a-5p/CXCL17	(44)
**non-TDEs**						
circEIF3K	↑	CAFs	CRC	Promote tumor proliferation, invasion and tube formation	miR-214/PD-L1	(45)
miR-126a	↑	MDSCs	BRC	Promote Th2 cell responses, tumor metastasis and angiogenesis	S100A8/A9, IL-33/IL-13	(46)
miR-155-5p	↑	TAMs	CRC	Promote tumor immune escape	ZC3H12B	(47)
miR-186	↓	NKs	Neuroblastoma	Inhibit tumor growth and immune escape	MYCN	(48)
miR-21	↑	CAFs	MDSCs	Promote MDSC accumulation, M-MDSC generation, and tumor cisplatin resistance	IL-6, STAT3	(49)
miR-3188	↓	CAFs	HNC	Inhibit tumor proliferation and apoptosis	BCL-2	(50)
miR-500a-5p	↑	CAFs	BRC	Promote tumor proliferation and metastasis	USP28	(51)
lINCEPHA6-1	↑	IFNβ-treated A549 cells	NKs	Promote NK cytotoxicity	hsa-miR-4485-5p/NKp46	(52)
LINC00273	↑	TAMs	LUAD	Promote tumor metastasis	NEDD4/LATS2, STAT3	(42)
lncAFAP1-AS1	↑	TAMs	Esophageal cancer	Promote tumor migration, invasion, and metastasis	miR-26a/ATF2	(53)
lncAGAP2-AS1	↑	TAMs	LC	Promote tumor radiotherapy immunity	miR-296/NOTCH2	(54)
lncNEAT1	↑	CAFs	Endometrial cancer	Promote tumor growth and doxorubicin resistance	miR-26-a/b-5p-YKL-40	(55)

BRC, breast cancer; BCa, bladder cancer; BCL2, B-cell lymphoma 2; CAFs, cancer-associated fibroblasts; CRC, colorectal cancer; EC; endometrial cancer; ESCC, esophageal cell squamous carcinoma; HCC, hepatocellular carcinoma; HNC, head and neck cancer; LC, lung cancer; LUAD, lung adenocarcinoma; NKs, natural killer cells; MDSCs, myeloid-derived suppressor cells; Th 17, T helper 17; TNBC, triple-negative breast cancer. ↑, upregulated; ↓, downregulated.

### The Functions of Exosomal ncRNAs in T Cells

T cells form their unique T cell receptor (TCR) and perform a variety of biological functions. Within the TME, in particular, a few crucial T cell populations significantly influence tumorigenesis and progression. Tumor-specific T cells are a vital part of the adaptive immune system, and the vast majority of T cells depletion within the TME will trigger tumor immune envision and immunosuppression (56).

**Figure 4:**
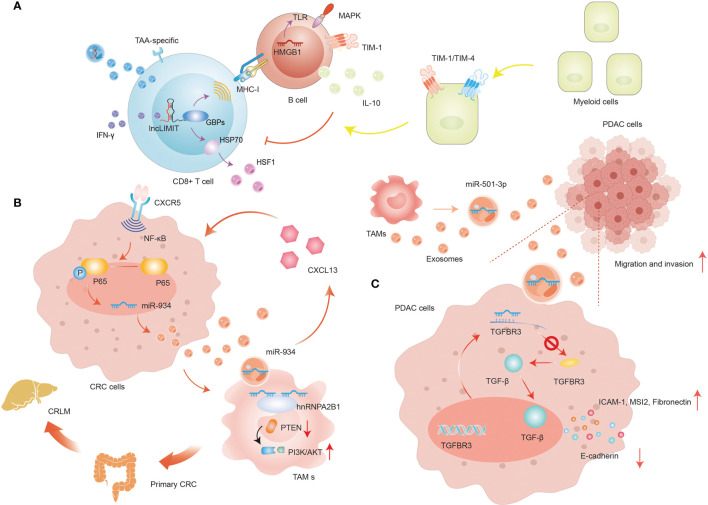
The regulation of exosomal ncRNAs in immune cells. **(A)** The regulation of exosomal lncRNA LIMIT in T cells. **(B)** The positive-feedback loop of CXCL13/CXCR5/NF-κB/p65/miR-934 in CRC cells. **(C)** The role of exosomal miR-501-3p in PDAC cells.

It is well known that programmed death-1 (PD-1) and its ligand PD-L1 are imperative immune checkpoint molecules involved in tumor immune invasion (57). PD-1 is a key receptor displayed on activated T cells and once PD-1 is mobilized, it prevents T cells from directing immune responses by reversibly suppressing T cell activation and proliferation (58). Intriguingly, non-small cell lung cancer (NSCLC) cells inactivate CD8+ T cells *via* delivering PD-L1-containing exosomes *in vitro* culture systems. In-depth investigations elucidate the detailed molecular mechanism by which circ-CAP4 inhibits PD-L1 inside and outside of the cell through binding to let-7 miRNA. intracellular PD-L1 governs NSCLC cell proliferation, metastasis, cisplatin resistance, and CSCs properties, while extracellular PD-L1 provokes the deactivation of CD8+ T cells to avoid immune clearance (59). In addition, ER stress is capable of dysregulating anti-tumor immunity *via* promoting the secretion of miR-23a-3p-rich exosomes from hepatocellular carcinoma (HCC) cells. Exosomal miR-23a-3p decreases PTEN transcriptional level and launches the AKT pathway, therefore elevating PD-L1 abundance in macrophages, and ultimately suppressing T cell functions (60). In CD8+ T cells, it has been identified LIMIT (lncRNA Inducing MHC-I and Immunogenicity of Tumor) is a novel immunogenic lncRNA, which is sensitive to IFN-γ. LIMIT engages in the modulation of IFNγ-induced MHC-I degree without modifying overall IFN-γ signaling (61). Distinctively, MHC-I is markedly ascended by LIMIT, whose abnormal expression is modulated by TAA-specific CD8+ T cell-mediated anti-tumor ability. TDEs are identified as possessing surface MHC-1, and thus potentially directly activate CD8+ T cells against tumors (62). Due to the excellent spatial proximity to GBPs, LIMIT elevates the transcription of GBPs in a cis-regulatory manner, stimulating CD8+ T cell priming and activation (63). Thus, serving as a cis-acting lncRNA, LIMIT fosters MHC-I assembly by energizing GBPs, which are transduced with HSP90 to release HSP90-associated HSF1 and enhance cancer immunity. Although numerous therapy strategies targeting HSP90 have been developed in clinical settings, no effective HSP90 inhibitor has been widely accepted into clinical applications so far. Considering the intimate interaction between GBPs and HSP90 as well as the unleashing of HSP90-induced HSF1 without adjusting HSP90 animation, targeting GBPs appears to be an attractive and potent strategy to modify HSF1 for anti-tumor immunity treatment. Delivery of lncRNA LIMIT-containing exosomes is capable of achieving this goal, and their advantages and convenience further boost the development of GBPs-based therapies (**Figure 4A**).

CD8+ T cells have been recognized as effective fighters defending against tumors; in contrast, CD4+ T cells intend to elicit a sustained immune response. Every nascent CD4+ T cell clone possesses the potential to differentiate into one of the separate subtypes (e.g., diverse T helper cells), thereby orchestrating different immune reactions in the context of the TME (64). This is essential because abnormal or inappropriate CD4+ T cell responses may result in the initiation and development of a primary tumor. In nasopharyngeal carcinoma (NPC), miR-24-3p is profoundly upregulated in TDEs that can be delivered into T cells, where it then inhibits the percentage of CD4+ T cells and decreases the production of IFN-γ and IL-17 while provoking the augment of CD4+ Foxp3+ T cells (Tregs) (65). Further investigations indicate that exosomal miR-24-3p has the potential of occupying the 3’-UTR regions of FGF11, which is responsible for followingCD4+ T cell degradation. Interestingly, the abundance of miR-24-3p in NPC is positively related to the frequency of CD4+ and CD8+ T cells and a dismal prognosis. Moreover, hypoxia upregulates miR-24-3p expression in exosomes, thus elevating the transcriptional level of P-ERK, P-STAT1, and P-STAT3, while reducing the abundance of P-STAT5 in CD8+ and CD4+ T cells.

Tregs, characterized by their exclusive cell surface molecules (CD25 and CTLA-4) and nuclear marker FoxP3, are a captivating subpopulation of T cells frequently involved in coordinating autoimmunity and homeostasis (66). CRC cell-derived exosomal miR-208b dramatically facilitates Tregs amplification by directly blockading the 3’-UTR regions of PDCD4 mRNA. PDCD4, a previously disregardful tumor suppressor gene engaged in programmed cell death, is strongly required in negatively administering Tregs expansion (67, 68). Moreover, mass transfer of miR-208b into Tregs shackles PDCD4 abundance and is positively associated with CRC oxaliplatin resistance (69).

Distinguishingly, it has been proposed that γδ T cells can exert immunosuppressive effects on a wide range of cancers (70). With the advancement of immune-oncology, γδ T cells have joined the “T cell arsenal” of anti-cancer. Nevertheless, the accurate subpopulation of γδ T cells and their regulatory mechanisms in BRC are not yet fully understood. Ni et al. found increasing numbers of infiltrating γδ1 T cells and identified CD73+γδ1 T cells are new Tregs in BRC. Further studies revealed that CD73+γδ1 T cells represent the primary Tregs in BRC and play an immunosuppressive role in dampening BRC progression through targeting adenosine (71). lncRNA SNHG16 is available to be delivered into tumor-infiltrating γδ1 T cells *via* TDEs. Exosomal SNHG16 functions as a competing endogenous RNA (ceRNA) to upregulate CD73 expression in γδ1 T cells by sponging miR-16-5p, resulting in the depression of its downstream gene SMAD5 and the augmentation of the TGF-β1/SMAD5 pathway (71).

### The Functions of Exosomal ncRNAs in DCs

DCs, as professional antigen-presenting cells, is responsible for bridging the gap between human innate and adaptive immune responses, and play an anti-tumor role in tumor progression, although it can be reversed in the TME. When DCs are threatened by pathogens or receive activation signals, they tend to initiate the activation of pattern-recognition receptors [e.g., toll-like receptors (TLRs)] and move to secondary lymphoid structures within the lymph nodes where they modify naive B or T cells (72). Once the TLR expressed on DCs is stimulated, DCs are induced to augment the synthesis and release of pro-inflammatory cytokines and the expression of costimulatory molecules, which subsequently activate T cells and initiate immune responses (73). Lewis lung carcinoma (LLC) cells secrete a certain number of miR-21- and miR-29-containing exosomes. And the two exosomal miRNAs directly target TLRs to provoke pro-tumoral inflammation, rendering tumor growth and metastasis (74). A coexistence of miR-29a and cytokeratin has been identified in the tumor vortex. Notably, miR-29a is colocalized with macrophage marker F-11 rather than cytokeratin at the edge of LLC. miR-29a is mainly concentrated in macrophages and can directly target TLR7 and TLR8 of macrophages. Both miR-21 and miR-29a exhibit similar GU features (GUUG of miR-21 and GGUU of miR-29a) in nucleotide fragments 18-21, and the GU pattern is dominant in TLR-activated RNA33. By targeting TLR8, these two exosomal miRNAs dominate the initiation of NF-κB pathway to mediate TNF-α and IL-6 emitting, thus inhibiting tumor propagation and expansion (74). Among LLC cells transfected with LNA anti-miR-21/29a, only miR-29a is not managed to be eliminated and is available to orientate the lung tissues, which implies that exosomal ncRNAs may be employed as potent inhibitors against TLR family members. Interestingly, it has been proven that the expression of TLR4 exerts a significant anti-tumor effect in DC-based immunotherapy. In DCs, tumor-derived exosomal miR-203 reduces TLR4 expression and inhibits the release of downstream cytokines (e.g., TNF-α, crucial to DCs maturation, and IL-12, required for Th1 differentiation), thus promoting cancer immunosuppressive microenvironment (75). In mice, the volume of MHC II and costimulatory molecules in miR-155-deficient DCs have no intensity fluctuations (76). While such DCs are relatively sluggish to the T cell stimulus. Alternatively, in human monocyte-derived DCs (moDCs), exosomal miR-155 is observed to be significantly upregulated during the processing of moDCs maturation. LPS exhibits great suppressive effects on the translation and cohesion of exosomal miR-155 by incompletely occupying its recognition region within the 3’-UTR (77). Further research demonstrates that a number of typical pathways are mutated in miR-155 knockdown LPS-activated moDCs, where IL-1 and its signaling components are involved in most of the detected signaling. Thus, by targeting TAB2, miR-155 can act as an important component of feedback negative regulator that downregulates the synthesis and release of inflammatory cytokine production through TLR4/IL-1 signaling, which also communicates in tandem with the p38 MAPK pathway.

Also, TDEs can serve as vehicles to deliver exogenous miR-155 into DCs, thus enhancing T cell proliferation and elevating LPS expression levels (78). Exosomal miRNA-155 facilitates DC maturation, which in turn enhances T cell proliferation and elevates DC IL-12p70 and IFN-γ expression. Interestingly, these DCs are more capable of activating the proliferative capacity of lymphocytes and mediating naive T cells’ differentiation into Th1 cells (79). miR-148 and miR-152 are overexpressed in TLR-activated DCs that decrease TLR-induced cytokine (e.g., IL-6, IL-12, and TNF-α) production and restrict Ag-specific CD4+ T cell clone and expansion. Also, these two miRNAs can directly inhibit CaMKIIa to mediate the upregulation of MHC II levels and the secretion of related cytokines in DCs (80).

### The Functions of Exosomal ncRNAs in Macrophages

Macrophages are one of the most prevalent and influential immune-associated stromal cell types in TME. Broadly, macrophages fail into two major phenotypes depending on the different responses of macrophages to specific stimuli: one is the classical inflammation-induced M1 macrophages, and the other is the alternatively-activated M2 macrophages, which are commonly designated as tumor-associated macrophages (TAMs) (81). Interestingly, TDEs have been demonstrated to induce macrophages differentiation into TAMs, therefore expediting tumor growth, angiogenesis, immune escape, and ECM rebuilding to facilitate locoregional and distant metastasis (82). Pancreatic ductal adenocarcinoma (PDAC) remains a recalcitrant and deadly disease with a dismal prognosis, and its incidence is still rising in recent years (83). However, remarkable progress has been made in understanding how exosome-mediated ncRNAs regulate the intercellular crosstalk in PDAC. For instance, an isolated study disclosed the exact roles of TAM-derived exosomal miR-501-3p during PDAC development (84) (**Figure 4B**). miR-501-3p decreases TGFBR3 expression by binding to the 3364-3370 bases of the TGFBR3-3’-UTR region. Moreover, it has been documented TGFBR3 plays an integral pro-tumorigenesis role in a wide range of tumors *via* the TGF-β signaling (85). Then, TGF-β diminishes the abundance of ICAM-1, MSI2, and Fibronectin, while promoting E-cadherin in TAMs *in vivo*. Knockdown of miR-501-3p in TAMs-derived exosomes partly shackles the transcriptional level of CSCs-related genes, eliciting PDAC metastasis and tube formation regression.

In PC, miR-301a-3p is significantly upregulated in exosomes and can be transferred into macrophages. By enhancing HIF-1a and HIF-2a expression, hypoxia elevates the expression level of miR-301a-3p both in PC cells and PC-derived exosomes. Knocking out miR-301a-3p in PC cells inhibits exosome releasing and polarization of the macrophages to an M2 phenotype by targeting PTEN/PI3K-signaling, ultimately curbing PC cell growth, invasion, migration, and EMT (86). Another recent study showed that, in PC, lncRNA TALC is largely expressed in TDEs. The relocated exosomal TALC in TAMs stimulates the M2 polarization of microglia. M2 polarization is associated with the excretion of the complement components C5/C5a. Exosomal lncRNA TALC stimulates p38 MAPK phosphorylation through targeting downstream ENO1. Functionally, C5 contributes to TMZ-mediated recovery from DNA damage, causing chemoresistance. And TALC-mediated TMZ resistance can be strongly limited by C5a-targeted to improve the effectiveness of immunotherapy (87). Intriguingly, two aberrant upregulated exosomal ncRNAs, miR-21 and miR-1246, which may change microglia and macrophages phenotype to enhance glioma proliferation (88, 89). lncSBF2-AS1 is exceptionally upregulated in glioma tissues, which is closely connected with temozolomide (TMZ) resistance (90). Also, SBF2-AS1 can be assigned into exosomes to relocate from chemotherapy-resistant glioblastoma (GBM) cells to chemotherapy-sensitive GBM cells, thereby transferring the capacity of TMZ resistance to recipient cells.

In CRC, miR-934 is markedly upregulated in tumor tissues and primary cells, in particular, in CRC liver metastasis (CRLM), and positively correlated with unfavorable prognosis (91). hnRNPA2B1 is able to mediate the sorting and wrapping of miR-934 into exosomes and dominate subsequent delivery of miR-934-containing exosomes to macrophages. Moreover, the exosomal miR-934 is implicated in inducing M2 macrophage polarization by regulating the expression level of PTEN and mobilizing the PI3K/AKT signaling pathway. Specifically, miR-934 downregulates PTEN abundance through binding to its 3’-UTR and stimulating the contents of p-AKT and p-PI3K in PMA-induced THP-1 cells. The invasive and liver-metastatic potential of CRC is closely related to the encouragement of TAMs, which are responsible for secreting chemokines and cytokines involved in the malignant transformation and more aggressive behaviors of tumors. And these regulatory molecules are presented in TDEs-polarized M2 macrophages (92). Further, miR-934 can dramatically elevate the release of CXCL13 in Kupffer cells. CXCL13 is a potent chelator that boosts accretion of CXCR5-expressing cells, while silencing CXCL13 and CXCR5 in CRC inhibits TAM-mediated metastasis, suggesting that exosomal miR-934 promotes CRLM progression by targeting the CXCL13/CXCR5 axis. Considering that the CXCL13/CXCR5 axis arouses the traditional NF-κB singling and exacerbates the inflammatory reactions in tumors, there might be a more elaborate regulatory mechanism of miR-934 that seems to be unexplored (93). Indeed, CXCL13 enhances p65 phosphorylation, increasing the level of NF-κB, MMP2, and MMP9 while decreasing IκBα. These alterations of CXCL13 can be reversed by helenalin, which exhibits great suppressive effects on NF-κB/p65 pathway, indicating that miR-934 could also be positively manipulated by NF-κB/p65 pathway *via* a positive feedback loop. In-deep studies showed that p65 straightforwardly upregulates miR-934 level inside the -2002 and -1500 bp domains by combining a specialized sequence (GGAAATGCCT) between p65 and miR-934 promoter. And the abnormal of the aforementioned specialized sequence eliminates the facilitative effect of CXCL13 and the inhibitory effect of NF-κB/p65 pathway on miR-934 enrichment. Taken as a whole, polarized M2 macrophages trigger pre-metastatic niche arrangement and foster CRLM by excreting CXCL13 and actuating a positive-feedback loop of CXCL13/CXCR5/NF-κB/p65/miR-934 in CRC cells (**Figure 4C**).

### The Functions of Exosomal ncRNAs in NKs

NKs are one of the major subsets of T cells in the innate branch of immunity. NKs commonly patrol the circulatory system, hunting for cancer cells. Among all the immune guardians, NKs are one of the most aggressive, with a potent ability to kill transplanted and spontaneous tumors. They are capable of producing a series of anti-tumor cytokines (e.g., IFN-γ), which not only influences target cells but also mobilizes T cells and macrophages to destroy tumor cells or enhance the anti-tumor vitality of other immune cells (94). To date, the exact roles of exosomal ncRNAs in NK biology have been controversial. TIM-3 is a vital co-inhibitory molecule expressed on NKs. Peripheral NKs from cancer patients tend to exhibit excessive expression of surface TIM-3, which is correlated with dysfunction and impairment of NKs (95). In HCC, circUHRF1 is upregulated in TDEs and it can increase the level of TIM-3, therefore impeding the physiological functions of NKs (38). Previous investigations have confirmed that circRNAs are primarily considered miRNA decoys, which attach to miRNAs and in turn boost the transcription of their target genes. Correspondingly, it has been identified that circUHRF1 can function as a targeting platform for miR-449c-5p, which attenuates the production of IFN-γ and TNF-α in NKs. Interestingly, there appears to be reciprocal regulation between circUHRF1 and miR-449c-5p in NKs. TIM-3 mRNA is a target of miR-449c-5p. It is notable that both permeating NKs and CD8+ T cells within the TME exhibit enrichment of TIM-3 (95, 96). These results show that there exist a circUHRF1/miR-449c-5p/TIM-3 axis in NKs and CD8+ T cells, denoting a novel mechanism of circRNA in the coordination of these two immunocyte types. However, the regulatory axis described above is unique in NKs and not present in CD8+ T cells. Forced exosomal circUHRF1 expression limits the susceptibility of HCC cells to anti-PD1 therapy. For another thing, the TIM-3/Gal-9 pathway is now well-established in NKs, therefore, PD1/PD-L1 and TIM-3/Gal-9 restriction may enhance the efficiency of NKs in immunotherapy (97). circUHRF1 in TDEs promotes TIM-3 expression, triggering NKs exhaustion. As a consequence, the height of circUHRF1 plays a decisive role during the anti-PD1 treatment of HCC. In neuroblastoma patients, miR-186 is lowly expressed in tumor cells and positively associated with clinical severity and prognosis outcome (48). miR-186 directly identifies the target sequence and impedes the translation of TGFBR1 and TGFBR2. Also, miR-186 is observed to be capsulated into NK-derived cytotoxic exosomes and act on MYCN, AURKA, as well as the TGF-β pathway (98). Exosomal miR-186, in particular, inhibits the cytotoxicity of NK by downregulating TGFBR1- and TGFBR2-induced suppression of TGF- signaling. That is, delivering miR-186 to the tumor site not only limits neuroblastoma cell growth but also independently counteracts the TGF-mediated immunosuppressive mechanisms that hinder the efficacy of ADCC-based treatment approaches. Intriguingly, killing molecules (e.g., perforin 1) are significantly reduced in exosomes originated from TGF-β-treated or growth factor-starved NK cells, implying that miR-186 (and possibly other exosomal RNA molecules) may have a unique impact on regulating the cytotoxicity of NK-derived exosomes. In addition, an increase in CD73 expression is detected in NKs, and the frequency of these CD73+ NKs are associated with larger tumor size in BRC patients (99). By acting on actin polymerization-dependent exocytosis, NKs are able to deliver CD73 from intracellular vesicles to cell surfaces and extracellular spaces upon contacting with 4-1BBL on cancer cells. Undergoing transcriptional reprogramming, these CD73+ NKs enhance IL-10 synthesis through STAT3 transcriptional activity, which subsequently inhibits CD4+ T cell proliferation and IFN-γ generation (99). This finding suggests that each immune cell population does not exist in isolation in the TME; instead, abundant information exchange between NK cells and other immune cells in TME. And such interactions largely depend on ncRNAs-containing exosomes shuttled in the TME.

### The Functions of Exosomal ncRNAs in CAFs

CAFs account for a considerable proportion of the stromal cells within TME and are an integral contributing factor in the generation of an aberrant TME that facilitates cancer development and progression. According to recent findings, CAFs are capable of regulating the biological properties of tumor cells and other stromal cells through intercellular communication. Specifically, CAFs alter and remodel the structure of the ECM that initiates tumor cell invasion. Also, they can interact with themselves or alternative TME cells by synthesizing and releasing cytokines, growth factors, and chemokines (100). Previous research has indicated that CAFs and tumor cells contact not only *via* traditional paracrine signaling pathways (such as the previously mentioned cytokines and chemokines) but also *via* exosomes (51). A study by Dou et al. used miRNA profiling assays and found abnormal expression of miR-92 isolated from CAFs exosomes (101). Exosomes-treated BRC cells exhibit higher levels of miR-92 and PD-L1 (102). miR-92 can directly bind YAP1 to promote YAP1 phosphorylation and consequently block YAP1 nuclear translocation. It is intriguing that YAP1 activity has been purported to modulate PD-L1 expression in certain types of carcinoma (103). Using ChIP, it has been verified that YAP1 occupies the enhancer region of PD-L1 to upregulate PD-L1 expression. Furthermore, CAF-derived exosomes increase PD-L1 expression, significantly enhancing T cell apoptosis and impairing T cell proliferation (101). Ren et al. identified the precise mechanism of Wnt/β-catenin signaling activation through CAF-derived exosomes by transfer of exosomal lncH19 (104).

### The Functions of Exosomal ncRNAs in MDSCs

MDSCs are immature, pathologically activated neutrophils and myeloid cells that are phenotypically and morphologically similar to monocytes. The immunosuppressive activity of MDSCs empowers them to modulate the immune response in numerous cancers. In human and mouse peripheral blood, MDSCs have been identified as immature cell populations with the capability to inhibit T cell activation and aggregation of MDSCs *in vivo* and *in vitro* (105). Jiang et al. discovered that the progression of early-stage MDSCs (eMDSCs) in BRC with elevated IL-6 expression is closely related to the SOCS3 deficiency-dependent overactivation of the JAK/STAT signaling (106). Both miR-9 and miR-181a are significantly overexpressed in eMDSCs, which not only facilitate the amplification of immature eMDSCs but also suppress the immunity of T cells. In addition, exosomal miR-9 and miR-181a can promote tumorigenesis and immunosuppression by enhancing *in situ* infiltration of eMDSCs. Nevertheless, the intrinsic mechanisms underlying the two miRNAs are different. miR-9 and miR-181a can induce the development of eMDSCs by diminishing SOCS3 and PIAS3 (two key regulators in JAK/STAT signaling pathway), respectively (106). In glioma, exosomal miR-1246 is shown to stimulate the activation and differentiation of MDSCs in a DUSP3-dependent or ERK-dependent manner. More importantly, the high exosomal miR-1246 in cerebrospinal fluid of postoperative patients is strongly associated with decreased survival and increased tumor recurrence (107). It is generally accepted that hypoxia is a critical feature of the GBM microenvironment. Hypoxia enhances the transcriptional and selective packaging of miR-1246 by upregulating POU5F1 and hnRNPA1, increasing miR-1246 expression in TDEs (108). Furthermore, the mechanism by which 2-methoxyestradiol, a microtubule inhibitor presently enters pre-clinical trials and clinical research in GBM, inhibits MDSC activation by suppressing the expression of the hypoxia-derived exosomal miR-1246 in tumor cells and PD-L1 expression in MDSCs has been identified (107).

### The Functions of Exosomal ncRNAs in B Cells

B cells are dedicated immune cells charged with antibody generation, processing of antigens and their presentation, and proinflammatory cytokine release. In general, B cells mainly reside in the periphery of tumor and are normally presented in lymph nodes adjacent to the TME. Similar to formerly discussed T cells, there exists a regulatory subtype in the B cell population known as regulatory B cells (Bregs) that is indispensable for tumor immunomodulatory within the TME (109). The role of T cells in tumor immunosurveillance had received a great deal of research attention. But only a few studies are concerning the functional characteristics of B cells in the TME. For example, an isolated study found that in HCC, miRNA HMGB1 plays a pro-tumorigenic role in infiltrating TIM-1+ Bregs (110). TIM-1 is an important transmembrane glycoprotein displayed on B cells as a unique biological label for Bregs (111). In HCC, Bregs secret large amounts of IL-10 and manifest eminently inhibitory effects on CD8+ T cells (**Figure 4A**). And such suppressive reaction can be further enhanced by myeloid cells by TIM-1/TIM-4 signaling. In addition, sufficient HMGB1-containing TDEs trigger TIM-1+B cell clones *via* targeting the TLR/MAPK signaling pathway. Mechanistically, activating TLR2/4 markedly augments the percentage of TIM-1+ B cells and heightens the negative roles of TDEs in CD8+ T cell breeding and TNF-α and IFN-γ procreation (110).

B cells are capable of forming a variety of complex tumor-associated immune aggregates, spanning from disorganized irregular small clusters to regular tertiary lymphoid structures (TLS), which supports further maturation and subtype conversion of tumor-specific B cells and the progression of tumor-specific T cell responses (112). In particular, antigen-specific reciprocal relationships betwixt T and B cells appear to be integral in TLS, and the antitumorigenic effects of T cells in the TME are largely dependent upon collaboration with B cells (113). Apoptotic exosome‐like vesicles (AELVs), emanated in a caspase-3-dependent way, are observed to induce TLS arrangement, thus facilitating autoimmunity and allograft inflammation (114). And such accommodative dysfunction is commonly accompanied by elevated CD20+ B cells density across the vessel wall and augmented CD3+ T cells infiltration in the intimal regions (115). Furthermore, AELVs provoke the accumulation of γδTh17 cells (a subset of CD3+ cells) to the allograft and foster the formation of γδT dependent TLS, while regulating IL-17 generation other CD3+ T cells (except γδTh17 cells) in the allogeneic organs *in vivo*.

The exosomal ncRNAs that are involved in the TME and determine susceptibility to tumor cells in T cells, DCs, and macrophages have been researched extensively over the past years, while similar studies on B cells are still scarce. One tantalizing research topic concerning exosomal ncRNAs in the TME is their precise regulatory machinery and biological significance in B cells.

### The Functions of Exosomal ncRNAs in Cancer Stem Cells

Within the tumor tissue, there exists a small group of cells with extremely high tumorigenic capacity and very low differentiation, called cancer stem cells (CSCs). Similar to the typical undifferentiated cells in tissues, CSCs represent the properties to perform self-regenerate and differentiation (116). They are indispensable for cancer progression and are the root causative factors of tumor recurrence, metastasis, and drug resistance. Nowadays, the presence of CSCs has been demonstrated in tumors of different tissue origins, such as BRC, CRC, and HCC (117).

It has been demonstrated that exosomal ncRNAs emerge as a novel layer affecting CSC characteristics. For example, lncRNA FMR1-AS1 is abundant in exosomes that can be released into the TME. lncFMR1-AS1 can bind to TLR7, activate the TLR7/NF-κB signaling, and elevate c-Myc expression, therefore promoting esophageal squamous cell carcinoma (ESCC) proliferation, anti-apoptosis, and invasive capacity (118). Intra-tumor hypoxia has been shown to be one of the main features of TME (119). Hypoxic bladder cancer cell-derived exosomal lncRNA UCA1 enhances CSCs proliferation and induces EMT to facilitate tumor metastasis (9). Ren et al. found that CAFs promote CRC cell stemness by emitting lncRNA H19-containing exosomes that activate Wnt/β-catenin signaling (104). This observation further confirms that tumor cells preferentially reside in the vicinity of stromal myofibroblasts, which exhibit higher Wnt signaling activity (120).

Emerging evidence implicates that CSC-derived exosomal ncRNAs play an essential role during tumorigenesis and tumor progression. Glioma stem cells (GSCs) are closely related to radiation and chemotherapy resistance as well as high recurrence rates of gliomas (121). An initial study reported that miR-944 levels are significantly decreased in high-grade gliomas, and the ectopic expression of miR-944 was associated with a dismal prognosis (122). Furthermore, the GSC-derived exosomal miR-944 dramatically limits glioma angiogenesis by targeting the 3’UTR of VEGFC, reducing VEGFC expression, and blocking the AKT/ERK signaling. Due to the self-renewal and drug resistance of CSCs within tumor tissues, novel effective therapies currently need to be developed. These new therapeutic interventions should not only have intrinsic anti-tumor activity but also the ability to kill CSC subpopulations. In BRC cells, RAB27B has been observed to facilitate the delivery of exosomes from stromal cells to BRC cells through transferring exosomal 5’-triphosphates. Exosomal 5’-triphosphates eventually stimulates the RIG-I signaling in recipient cells, resulting in the activation of IRDS genes. Simultaneously, activated RIG-I can act on the NOTCH3 signaling to muddy the extension of anti- tumor infiltrating cells in CSCs (123).

Of interest is the fact that the immune components of the TME are centered on the tumor cells and spread along the periphery, dynamically evolving with the stage of tumor progression. The various immune cells within the TME are not isolated from each other, instead, there is a colorful spatio-temporal dialogue among them. In addition, they create this complex network together with tumor cells, and stromal components. Also, exosomal ncRNAs play an indispensable role in the contact among TME compositions. Thus, exploring the underlying mechanisms of the TME and identifying the precise target exosomal ncRNAs of intervention can improve the therapeutic efficacy of cancers.

## Exosomal ncRNAs Regulate Tumor Progression in the TME

### Exosomal ncRNAs Control Tumor Angiogenesis in the TME

An enormous of research has unveiled that exosome is intrinsically connected with tumor angiogenesis (124). Nowadays, evidence is accumulating that exosome executes its angiogenic regulatory roles partially by delivering exosomal ncRNAs in the TME (125). Therefore, elucidating the underlying molecular mechanisms correlated with tumor angiogenesis is imperative to advance modern anti-angiogenic therapies.

One of the major pathways in which exosomal ncRNAs are engaged in tumor angiogenesis is through shackling the vascular endothelial growth factor (VEGF) and its receptor family. VEGF interacts with tumor angiogenesis by adjusting ECs arranged on blood vessels in an army of ways. In neighboring normal bronchial cells, lung cancer (LC) cell-derived exosomal miR-21 induces VEGF biosynthesis and angiogenesis progression in a STAT3-dependent manner (126). It was observed that miR-21 overexpression augments the expression of HIF-1 and VEGF, which in turn enhances the AKT and ERK1/2 signaling pathways, leading to an intensification of tumor angiogenesis (127). Exosomal circSHKBP1 is notoriously elevated in GC cells and is reported to aberrantly promote VEGF secretion and accelerate angiogenesis through the attachment of miR-582-3p, thereby enhancing HUR expression and improving VEGF mRNA cohesion (128). In chondrosarcoma cells, lncRNA RAMP2-AS1 is rich in TDEs. Simultaneously, exosomal lncRAMP2-AS1 functions as a ceRNA for miR-2355-5p to coordinate the expression of VEGFR2, therefore driving the angiogenic ability of human umbilical vein endothelial cells (HUVECs) (129). Likewise, PC cell-derived exosomal lncUCA1 is reported to be able to be transferred to and internalized by HUVECs, which may provide a novel paradigm for tumor angiogenesis (130).

In addition to targeting VEGF, tumor-derived exosomal ncRNAs can promote angiogenic potential by acting on other membrane and intracellular molecules. miR-9 directly targets MDK and regulates the PDK/AKT pathway to accelerate tumor angiogenesis (131). Hypoxia is another factor engaged in angiogenic proceedings that may influence the movement of various substances and manipulate the expression of exosomal ncRNAs. Under hypoxic conditions, LC cells produce quantities of exosomes in which miR-23a is strongly upregulated and restricts the action of prolyl hydroxylases 1 and 2, leading to the assembly of HIF-1 α in ECs and thus angiogenesis in LC cells is further enhanced (28). circ100338 is abundant in HCC-derived exosomes that can be transferred into recipient ECs to enhance the proangiogenic activity of HCC cells (132). Despite being the subject of intensive research, studies on the explicit functions of exosomal ncRNAs in tumor angiogenesis are yet in their infancy, which drives the need for further investigation.

### Exosomal ncRNAs Modulate Tumor Metastasis in the TME

As a hallmark of malignancy, metastatic dissemination is the leading cause of more than 90% of cancer-related deaths and is currently an insurmountable problem for curative therapies (133). Evidence is accumulating that the intercellular interactions among TME cells are a pivotal element of metastatic potential and organ-specific metastasis. However, recent studies are indicative of the vital roles of TDEs in modifying the microenvironment of both local and distant non-tumor cells. Furthermore, the construction of TDEs creates a favorable microenvironment for future metastatic sites, allowing tumor cell growth, expansion, and dissemination through augmented vascular permeability and metastasis of distant organs in a non-random metastatic pattern (134, 135). And the determinants of exosome-mediated organ-specific regulation render a change in the direction of metastasis. Specific integrins expressed on TDEs are distinctive, which defines the adhesion of exosomes to different cell types and ECM fragments in distinct organs (136).

Many groups have demonstrated that exosomal ncRNAs fuel initial tumorigenesis and endow tumor cells with metastatic properties. lncRNA HOTAIR is enriched in LC-derived exosomes. Its anomalous expression dramatically accelerates LC cell growth, invasion, and metastasis (137). It is worth noting that miR-30a-5p is plentiful in exosomes originating from vascular ECs that can be transported into lung adenocarcinoma cells to promote tumor malignant progression by targeting CCNE2 (138). In bladder cancer, exosomal lncLNMAT2 is observed to be capsulated by directly interacting with hnRNPA2B1. LNMAT2 is then recognized and taken up by HLECs, thereafter augmenting the transcription level of PROX1 *via* recruiting hnRNPA2B1 to its promoter, ultimately leading to lymphangiogenesis and lymphatic metastasis (139). Moreover, research has established that upregulation of circIARS in PC patients was positively correlated with tumor metastasis as well as unfavorable survival (140). IARS specifically targets miR-122 in HUVECs to escalate RhoA, leading to an increase in RhoA expression level and activity, thus further promoting ZO-1 expression and inducing F-actin expression. Subsequently, IRAS is internalized into HUVECs *via* TDEs, thereby enhancing the penetration of endothelial monolayer and thereby exacerbating tumor infiltration and dissemination.

### Exosomal ncRNAs Regulate EMT in the TME

EMT is generally defined as a complex biological process engaged in tissue reconstitution and has been extensively investigated as a promotor of tumor invasion and metastasis. Recently, considerable research efforts have been dedicated to achieving a comprehensive understanding of the exact functions of exosomal ncRNAs in modulating EMT (141, 142). During cancer development, activation of EMT grows the percentage of a mesenchymal phenotype related to highly invasive tumor cells that infiltrate the circulatory and lymphatic systems and reach distant sites for settlement. A recent study identified several miRNAs, including miR-193a-3p, miR-210-3p, and miR-5100, with altered levels in the exosomes of hypoxic bone marrow mesenchymal stem cells (BMSCs) that are observed to be transferred into epithelial cancer cells to activate STAT3 signaling and elevate the expression of mesenchymal-associated molecules, thereby promoting EMT progression and initiating tumor metastasis (143). Moreover, Duo et al. reported that the CRC-derived exosomal miR-27b-3p was upregulated in EMT and that it could increase the permeability of blood vessels by targeting p120 and VE-cad (144). p120 is required in the formation of VE-Cad cytoplasmic territory and expedites the stabilization of VE-Cad, which is indispensable for the establishment of adherent junctions owing to its ability to preserve junctional integrity and limit unrestricted vascular growth (145). miR-27b-3p inhibits ovarian cancer angiogenesis mimicry by acting on VE-Cad. In HUVECs, exosomal miR-27b-3p suppress VE-Cad and p120 expression, which accordingly leads to increased vascular permeability. Exosome-mediated linc02470 and linc00960 in high-grade bladder cancer cells induce tumor EMT by upregulating Notch signaling, β-catenin signaling, and Smad2/3 signaling (146). Mechanistically, Notch signaling stimulates the DNA-binding capacity of NF-κB, which elevates MMP9 expression. The MMP9 can reshape the ECM and promote the extravasation of tumor cells (147). Smad complex binds to regulatory elements and induces transcription of crucial genes correlated with EMT (148). Active Smad2 facilitates invasion of mesenchymal spindle cancer cells while suppressing E-cadherin expression (149).

### Exosomal ncRNAs Mediate Tumor Drug Resistance in the TME

Chemotherapy, or chemotherapy-based combination treatment, appears to be one of the most widely used cancer therapies, but the current carcinoma research field remains plagued by a major obstacle: chemotherapy resistance, which restricts the efficacy of the cancer treatment. Various molecular mechanisms have been associated with drug resistance, including markedly ascended levels of drug efflux and target mutations (150). Exosomes, as fundamental communicators of cell-to-cell crosstalk, are engaged in the formation and transmission of drug resistance. As a hot topic of research in recent years, exosomal ncRNAs are emerging as an imperative factor during the transmission of tumor drug resistance. Therefore, identifying the underlying adjustment paths of exosomal ncRNAs in tumor chemoresistance is indispensable for optimizing current therapeutic strategies.

It has been found that several cell membrane transporter proteins facilitate the efflux of drugs as a means of conferring resistance to chemotherapeutics that are commonly used. Most notably, the transmembrane proteins of the ATP-binding cassette (ABC) transporter family regulate the flow of diverse unrelated chemotherapy drugs across the cell membrane (151). The ABC transporter increases drug efflux, which is one of the mechanisms of drug resistance in tumors. Oxaliplatin resistance is currently an insurmountable problem for patients with advanced-stage CRC. Oxaliplatin resistance is currently an insurmountable problem for patients with advanced-stage CRC. Oxaliplatin-resistant CRC cell-derived exosomal circ0005963 (also termed as ciRS-122) can be transferred into oxaliplatin-sensitive cells, where it hastens glycolysis by elevating PKM2 level, resulting in enhanced ATP production (152). It is widely accepted that solid tumor cells frequently bank on anaerobic oxygen glycolysis rather than aerobic oxidation for biosynthesis and ATP requirements despite the low efficiency of such energy production (153). Thus, sufficient energy could confer greater motivation to transporters to pump oxaliplatin out of cancer cells in an ATP-dependent way, reducing the maintenance period of drug efficacy and accelerating drug efflux (154). Cells overexpressing ABC transporters have multiple drug resistance phenotypes after undergoing EMT, which also affects cellular resistance to drug-induced apoptosis and subsequently modulates tumor resistance by influencing drug efflux (155, 156). Liu et al. have investigated the predominant concerning factors of exosomal miR-128-3p in tumor chemoresistance (157). In CRC, overexpression of miR-128-3p is conducive to DNA repair and IC50 reinforcement, as well as low apoptosis rates, illustrating miR-128-3p as a predicted candidate of responsiveness to oxaliplatin. miR-128-3p-loaded exosomes can be assigned to cancer cells, negatively handling the promotor of Bmi1 and MRP5, which participate in oxaliplatin-mediated EMT and chemical agent excretion by targeting TWIST1 and EZH2 respectively. Also, this high degree of 128-exo leads to increased tissue distribution and internalized drug localization, reprogramming destructive DNA-platinum polymer that subsequently eradicates cancer cells.

Drug resistance is also influenced by alterations in drug targets, such as mutations or changed expression levels. As previously discussed, in oxaliplatin-resistant CRC cells, miR-128-3p was able to alleviate chemosensitivity by regulating Bmi1 and MRP5 (157). The miR-522 present in serum is predominantly produced from CAFS-derived exosomes. Interestingly, hnRNPA1 can mediate the packaging of miR-522 into exosomes, and ubiquitin-specific protease 7 (USP7) improves the stability of hnRNPA1 *via* de-ubiquitination. Cisplatin and paclitaxel are shown to stabilize hnRNPA1 by activating the USP7/hnRNPA1 axis. This promotes the secretion of CAF-derived exosomal miR-522, leading to inhibition of ALOX15 and reduced lipid-ROS aggregation in tumor cells, thereby suppressing tumor chemosensitivity (11). Another study showed that hnRNPA1 can directly bind a specific sequence (UAGGUA) of miR-196a, thus packaging miR-196 into CAFs-derived exosomes. miR-196a is then transmitted into head and neck cancer (HNC) cells, wherein it targets CDKN1B and ING5 to increase cell proliferation and decrease apoptosis (158). These findings have significant implications for the understanding of how exosomal ncRNAs are involved in tumor resistance. Also, more efforts are required to investigate other mechanisms underlying tumor resistance.

## The Clinical Significance of Exosomal ncRNAs in the TME

**Figure 5:**
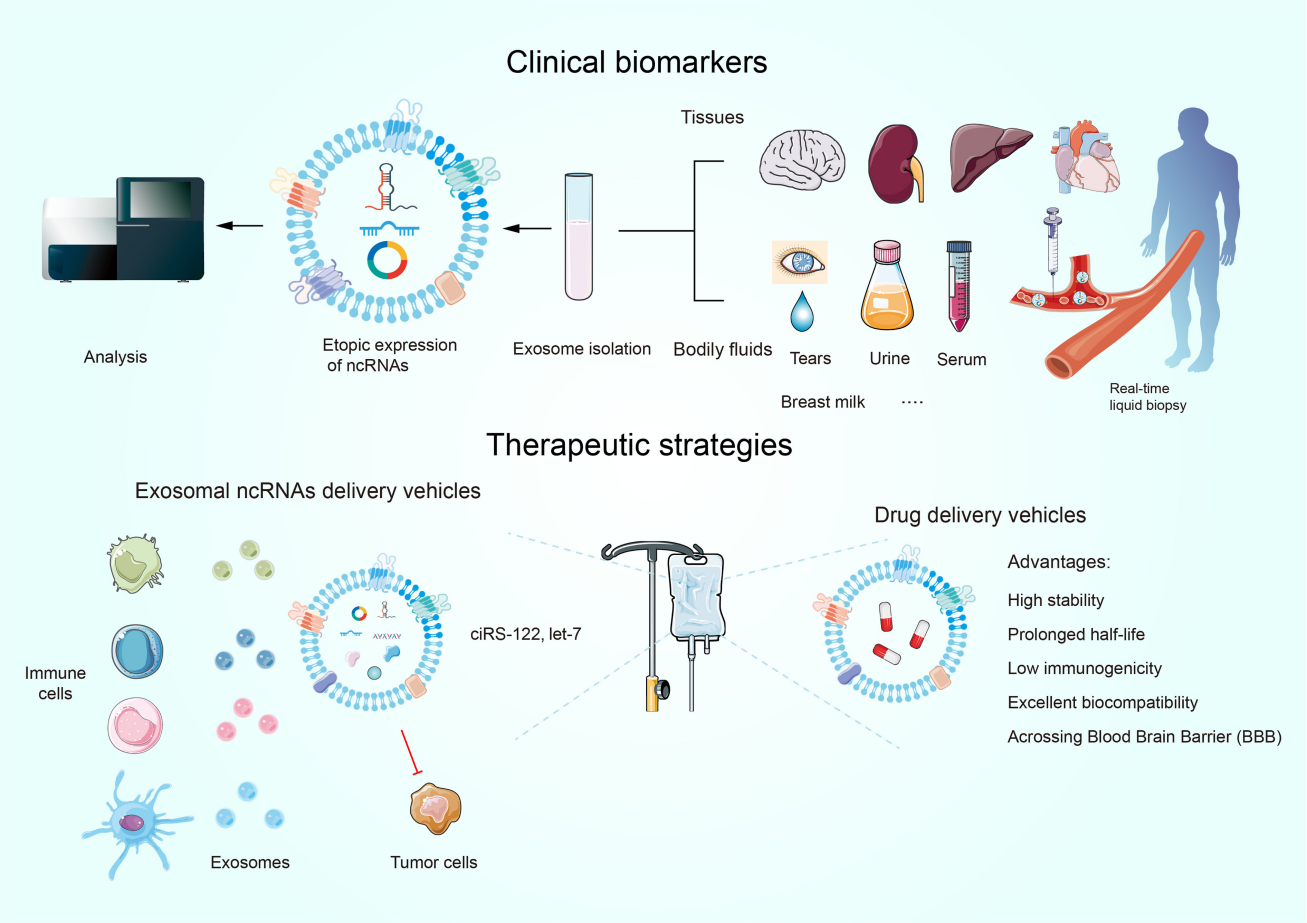
Exosomal ncRNAs as promising biomarkers for the diagnosis and prognosis of human cancers. Exosomes have novel therapeutic applications.

The promising role of exosomes as novel organelles in tumor diagnosis, prognosis, and therapy has attracted a wide range of attention. Exosome-mediated ncRNAs, unlike those unleashed straightforwardly into the circulatory system, are sheltered by a lipid bilayer, which can prevent ribonuclease destruction in a wide range of biological liquids (159). Additionally, they are highly mounted into exosomes in tumor cells and remain stable at different temperatures (160, 161). Given the merits of exosomal ncRNAs and the fact that exosomes are present in multiple bodily fluids (e.g., blood, urine, tears, and breast milk), as well as the specificity of the maternal cell cargo that produces exosomes and the conditions under which they are produced, there is a bright future for diagnostic and prognostic techniques based on exosomal ncRNAs (**Figure 5A**). An isolated study assessing the expression of GC tissue-specific and pre/post-operative plasma-specific miRNAs found let-7c expression was decreased while miR-21 and miR-106a expression were magnified in plasma. The ratio of miR-106a/let-7a expression serves as a biomarker for distinguishing GC (162). Urinary-specific exosomal circPRMT5 was significantly elevated in patients with bladder urothelial cancer, and high circPRMT5 expression intensifies lymph node metastasis (163). This suggests that urinary exosome-specific circPRMT5 is valuable in cancer diagnosis. Clinical studies have validated the enrichment of exosomal circUSP7 in NSCLC samples and upregulated USP7 predicts unfavorable outcomes and CD8+ T cell dysfunction, suggesting that it might be adopted in clinical arena to harness its clinical potential (39). Furthermore, the abundance of exosomal circUSP7 is not linked with TP53, KRAS, or ALK mutations, which have been confirmed as predictors in anti-PD1/PD-L1 therapy (164). Thus, detecting the level of exosomal circUSP7 appears to be a novel and appealing predictive biomarker in anti-PD1 immunotherapy in NSCLC. These discoveries help to classify patients to identify those who need follow-up for early diagnosis of recurrence and additional treatment.

Currently, drug delivery pattern dependent on nanotechnology represents one of the most potent vehicles to attain this intention. It is noteworthy that exosomes display a number of advantages over other nanoparticles owing to their good biocompatibility, high stability, extended half-life, low immunogenicity, precise targetability, and their ability to cross physical barriers (e.g., the blood-brain barrier) (**Figure 5B**) (165). Furthermore, the utility of exosomal ncRNAs as therapeutic tools calls for the ability of exosomes to deliver boundary ncRNAs to dysfunctional cells at both the primary research and clinical practice layers. For example, let-7a suppresses cancer progression by decreasing the levels of HMGA2 or RAS family members (e.g., KRAS) (166). GE11-positive exosomes, which contain let-7a, are used to inject intravenously into tumor-bearing mice by the tail-vein injection. Although numerous studies have demonstrated that let-7a does not appear to impact the expression of HMGA2 or RAS family members either *in vivo or in vitro*, GE11-positive exosomes specifically target EGFR-expressing cancer cells and restrain the growth of HCC70 BRC cells *in vivo* compared to let-7a-free and GE11-free exosomes (167). This suggests that let-7-free GE11 exosomes are a potential tool for delivering drugs to EGFR-expressing cancers such as BRC.

Alternatively, given their functions in mobilizing the migration and mediating the intercellular crosstalk of immunocytes, exosomal ncRNAs are tightly linked to both anti-tumor and pro-tumor immunoreactions, as well as the sensitivity and tolerance to tumor immunotherapies. Studies have shown that KRAS-induced exosomes play multifaceted roles in maintaining lung immunosuppressive metastasis and bring about novel routes for intractable metastasis suppression, especially in chemotherapy-resistant cancers. KRAS-induced exosomes independently drive the reprogramming of SMARCE1/NCOR1 chromatin genes, accelerating the construction of pre-metastatic niches in LC. In patients with metastatic LC, co-cultured with carboplatin primed RIP3/TNF-α-dependent necroptosis concomitant with a distinct abundance of immune inhibitor miR-146/miR-210 (168). It is of great interest to inhibit the level of KRAS-derived exosomes, thus enhancing the immune response to limit LC migration and dissemination. And this therapy might be successful with exogenous administration of exosomal miR-146, a crucial immunosuppressive regulator which is actively involved in RIP-3 dependent necroptosis. Through augmenting the PD-L1 expression in recipient cells, mesenchymal stem cells (MSCs)-derived exosomes (the main cargos are TGF-β, C1q, and semaphorins) dramatically contribute to the aggregation of CD206+ MDSCs and TAMs in the TME, leading to the accumulation of TGF-β and depletion of INFγ+CD8+ T cells (169). In contrast, GW4869-treated MSCs can reverse the alteration of PD-L1 expression and block the polarization of M2 macrophages. It is clear that the peculiar re-sculpturing of MSCs at cancer beds is responsible for the generation and release of exosomes, which are essential for building up the novel immune microenvironment into one that favors the escape of tumor cells from tumor-responsive T cells. This observation provides compelling evidence that targeting MSCs is a promising therapeutic strategy available to reinstate T cell-mediated anti-tumor cytotoxicity in patients with BRC. However, corresponding clinical trials that have not yet been conducted need to be carried out in future studies.

To date, considerable efforts have been devoted to investigating therapeutic strategies of exosomal miRNAs. However, only a few studies have addressed how to develop the therapeutic potential and value of exosomal circRNAs and lncRNAs. Oxaliplatin-sensitive cells respond trickly to the exosomal ciRS-122 from oxaliplatin-resistant cells, which endorses CRC glycolysis *via* enhancing PKM2 expression (152). Delivery si-ciRS-122 based on exosomes can significantly attenuate CRC glycolysis and rescue cell responsive sensitivity to oxaliplatin *in vivo*. HCC cells could react with circ0051443-containing exosomes from normal cells and reversely inhibit the self-malignant phenotype (170). Effective molecular inhibitors of cancer exosomal lncRNAs are still nascent and exploiting their specific tertiary structures (hairpin repeats or triple helices) may assart a new territory for the development of cancer treatment.

## Conclusion and Perspective

Studies involved in the interplay of exosomal ncRNAs and TME have become a hot spot worldwide. The body of work outlined here demonstrates a clear role for the exosomal ncRNAs in the extrinsic regulation of TME, including T cells, B cells, macrophages, NKs, CAFs, DCs, and MDSCs. Exosomal ncRNAs are crucial signaling molecules and biological messengers for dialogue among TME cells and play an indispensable role in angiogenesis, EMT, and metastasis of the TME. Notably, TDEs are quickly becoming an important element of a tumor-curated information network aimed at promoting tumor immune escape. The immunosuppressive molecules and factors secreted by TDEs affect immune cell functions such as anti-tumor activity. However, their regulatory mechanisms and functional characteristics are still in their infancy and need to be further explored. Indeed, there are a set of challenges that remain. First, stringent isolation of specific exosome populations is required since the numerous types of exosomes from diverse sources pose problems for detection when they sink into body fluids. One tantalizing possibility for the purification of exosomes is the single exosome assay technique to individually label exosomes that could examine fingerprinting characteristic information for each exosome. In turn, tumor-associated specific exosome subpopulations are identified through big data and artificial intelligence analysis and used as biomarkers for tumor warning signals. Second, is the feasibility of loading the desired cargo into exosomes. Although most efforts have been devoted to developing small-molecule inhibitors, exosomal ncRNAs appear to be emerging as a platform with much broader and complementary clinical implementations. For instance, ncRNAs could be skillfully incorporated into exosomes by handling the source cells to produce excessive ncRNAs cargoes. However, this process is difficult to achieve in terms of both technical means and experimental methods. Researchers failed to mount miRNA into HEK-293-derived exosomes with electroporation (171). Also, other methods of obtaining the desired exosomes may exist: i.e., by engineering specific inducers to boost the secretion of the desired exosomes, or by designing powerful agonists to potentiate the effect of the exosomes. Third, considering the large body of studies and clinical investigation work committed to pushing exosomal ncRNA-based immunotherapeutic methods, it is necessary to precisely quantitate exosomal ncRNAs and their underlying clinical toxicity. The fact that exosomal ncRNAs are involved in both pro- and anti-tumor effects reveals the complicatedness of the exosome system, as a given exosomal ncRNA may exert a double response in a temporal- or spatial-dependent manner during tumor progression. One exosomal ncRNA might serve as a tumor suppressor, and transmission of this ncRNA does provide a potential therapeutic strategy. Nevertheless, this ncRNA may also play an integral role in a wide range of biological processes, and its aberrant regulation would entail a destabilization of cellular homeostasis and therefore trigger severe secondary consequences. As a result, the comprehensive exosomal ncRNAs landscape in the TME needs to be taken into consideration in the milieu of the force of cancer immunity to ascertain their functions in the overall response. On the other hand, the distribution trend of specific exosomal ncRNAs could be correlated with susceptibility to immunotherapies and, as such, they are available to be utilized as encouraging biomarkers for surveying and forecasting the response to treatment.

Despite advances in the interaction between exosomal ncRNAs and TME, there is still a long way from basic research to clinical practice: exosomal ncRNAs as biomarkers and therapeutic targets. Therefore, comprehensive and cross-sectional research integrating multiple clinical data sets such as therapeutic significance will be needed to achieve this goal.

## Author Contributions

ZL collected the related paper. ZJ wrote the draft and revised it. JJ revised it. LY collected the tables and designed them. All authors contributed to the article and approved the submitted version.

## Conflict of Interest

The authors declare that the research was conducted in the absence of any commercial or financial relationships that could be construed as a potential conflict of interest.

## Publisher’s Note

All claims expressed in this article are solely those of the authors and do not necessarily represent those of their affiliated organizations, or those of the publisher, the editors and the reviewers. Any product that may be evaluated in this article, or claim that may be made by its manufacturer, is not guaranteed or endorsed by the publisher.
